# Moralised reputational governance in transnational higher education: relational aggression in Chinese female co-national peer networks

**DOI:** 10.3389/fpsyg.2026.1798940

**Published:** 2026-07-01

**Authors:** Wenfei Sun, Yajing Wang, Nabia Manzoor Shah Syed

**Affiliations:** 1 School of Marxism, Sichuan University, Chengdu, China; 2 School of Educational Studies, Universiti Sains Malaysia, Penang, Malaysia

**Keywords:** co-national peer networks, international students, moralised reputational governance, relational aggression, transnational higher education

## Abstract

**Introduction:**

This study examines how relational aggression functions as a form of informal regulation within Chinese co-national peer networks in transnational higher education, with particular attention to its processual and networked dynamics. Unlike conventional approaches that treat relational aggression as isolated interpersonal conflict, this study conceptualises it as a networked and processual phenomenon that shapes access to social belonging and peer-mediated resources.

**Methods:**

Drawing on qualitative interviews with Chinese female international students in multiple host-country contexts, the study adopts reflexive thematic analysis to explore participants’ lived experiences within transnational mobility trajectories.

**Results:**

The study develops and theorises the concept of moralised reputational governance. This concept illuminates how everyday social frictions are reinterpreted through shared moral codes, reconfigured into reputational cues, and enacted through indirect relational practices such as coalition alignment, narrative control, and information gatekeeping. These dynamics generate forms of harm that remain systematically less visible within institutional systems organised around discrete, evidence-based incidents, producing a structural legibility gap.

**Discussion:**

By linking micro-interactional processes with network conditions and institutional logics, the study advances a process-oriented theorisation of peer governance in transnational student contexts and contributes to research on international student experiences and higher education governance.

## 1 Introduction

China is the world’s largest source of international students (QS Quacquarelli Symonds, 2025; Yang, 2020). In major host countries such as the United Kingdom (UK), Malaysia and Australia, Chinese students constitute the largest international student groups (Ahmad, 2025; Men et al., 2024; Zhu and Sharp, 2021). Existing research has extensively documented the challenges these students encounter, including unfamiliar pedagogical practices, language barriers, acculturative stress, and uneven institutional support (Bi and Ahmad, 2024; Chen et al., 2021; Fu and Mok, 2025; Wu et al., 2025; Xiong et al., 2025). Within such contexts, co-national peer networks are widely recognised as important resources that provide access to information, emotional reassurance, and practical assistance, particularly early in mobility (Ahmad, 2025; Zhong et al., 2020).

However, this reliance is inherently ambivalent. While co-national networks can foster belonging and support (Alyafei and Malkawi, 2025; Laursen, 2021; Si et al., 2025), they may also reproduce intragroup stratification, peer monitoring, and exclusion (Dangal and Singh, 2020; Krajewski et al., 2025). In dense and relatively closed networks, inclusion and access to peer-mediated resources become contingent on reputational standing and network position (Cheng and Yu, 2024; Spencer-Oatey et al., 2017; Wang and Mireles-Rios, 2025). Although prior research acknowledges this ambivalence, it remains largely descriptive and offers limited insight into how supportive relations become routinised forms of informal regulation.

This study argues that these ambivalent and stratifying dynamics can be understood as a process of moralised reputational governance. Rather than treating relational aggression as isolated interpersonal conflict, the study conceptualises it as a mechanism through which everyday interactions are moralised, transformed into reputational cues, and enacted through indirect relational practices that regulate access to belonging and peer-mediated resources. In this sense, peer networks operate as evaluative arenas in which inclusion and exclusion are continuously negotiated through dispersed and often indirect forms of judgment.

Relational aggression provides a key interactional lens for examining this process. It refers to indirect, relationship-based practices such as exclusion, rumour circulation, and manipulation of social ties (Kyranides et al., 2024; Owens et al., 2000; Yue and Zhang, 2023). In practice, relational aggression is enacted through recurrent micro-practices such as exclusion, insinuation, alliance formation, selective communication, and information gatekeeping (Curtis et al., 2023; Patafio et al., 2025; Voulgaridou et al., 2022). These practices are typically subtle, dispersed, and plausibly deniable, making them difficult to contest (Kim et al., 2022; Voulgaridou and Kokkinos, 2023; Xiao et al., 2025). When enacted across repeated interactions within dense networks, such practices accumulate into patterned processes that shape reputational standing, access to resources, and participation.

This process unfolds through three interrelated mechanisms. First, everyday interactions are subject to moral coding, whereby ordinary behaviours are interpreted through shared moral vocabularies that render individuals evaluable (Fraser et al., 2022; Hegarty et al., 2022). Second, these evaluations are amplified through signal circulation, as interpretations travel across network ties and gain stability through repetition and uptake. Third, relational sanctioning translates reputational cues into patterns of inclusion, exclusion, and differential access to peer-mediated resources (Bakogiannis, 2026; Rousseau, 2026). Through these interconnected processes, relational aggression operates as a distributed and cumulative form of informal governance rather than as discrete interpersonal conflict.

For Chinese female international students, these dynamics are especially salient. Gendered expectations of relational propriety and culturally embedded concerns with face (mianzi) heighten sensitivity to peer evaluation and increase the perceived costs of confrontation (Ajah-Okohu, 2025; Ma et al., 2021; Zhu et al., 2024). Relational aggression is more prevalent in female peer interactions and tends to take indirect forms, whereas male conflict is more often direct (Crick et al., 1995; Underwood, 2003). Research on female peer relationships highlights the prevalence of relational aggression, such as gossip, exclusion, and reputational management (Cozza et al., 2022; Magram et al., 2025). In such contexts, attempts to contest exclusion may themselves be interpreted as deviance, constraining help seeking and rendering withdrawal or silence a rational strategy (Akaenyi, 2024; Benenson et al., 2013; Cristina, 2021; Owens et al., 2000). These responses should therefore be understood as situated forms of agency shaped by reputational risk and social norms.

Despite growing recognition of the ambivalence of co-national networks, three limitations remain. First, existing research rarely explains how evaluative attention becomes routinised boundary-making. Second, the production and stabilisation of reputational cues remain under-theorised. Third, insufficient attention has been paid to how cumulative and networked harms are recognised within institutional contexts, particularly given that formal systems are typically organised around discrete and evidence-based incidents. These limitations point to the need for a clearer, process-oriented framework that links interactional mechanisms, network dynamics, and institutional recognition.

Addressing these gaps, this study examines Chinese female international students’ experiences of relational aggression within co-national peer networks and investigates how cumulative harms become institutionally illegible. Drawing on qualitative interview data across multiple contexts, it develops a process-oriented account linking micro-interactional practices, network conditions, and institutional logics. Participants’ responses are analysed as forms of situated agency shaped by reputational vulnerability and structural constraints.

This study addresses the following research questions (RQ):

1. How is relational aggression enacted in Chinese co-national peer networks among international students?
2. How do Chinese female international students interpret and respond to relational aggression?
3. How do university reporting and support processes recognise and handle these experiences?
4. What consequences do these experiences have for students’ wellbeing, participation, and trajectories?

By integrating relational aggression research, social network theory, and institutional perspectives, this study advances a process-oriented conceptualisation of peer dynamics as moralised reputational governance. In doing so, it contributes to research on international student experiences and higher education governance by demonstrating how interactional processes, network structures, and institutional logics jointly shape patterns of inclusion, exclusion, and recognition.

## 2 Literature review

This review develops a theoretical foundation for understanding how peer relations in international student contexts operate as processes of informal regulation. It synthesises three strands of literature on co-national peer networks, relational aggression, and institutional recognition of harm. Rather than treating these strands as separate domains, the review brings them into analytical dialogue to show how interactional processes, network structures, and institutional logics jointly shape patterns of inclusion, exclusion, and recognition.

### 2.1 Co-national peer networks as evaluative infrastructures

Co-national peer networks are widely recognised as key support structures for international students, particularly in the early stages of transnational mobility (Al Ahmad et al., 2025; Compiegne, 2021; Zhong et al., 2020). These networks provide access to practical information, emotional reassurance, and culturally familiar forms of interaction, often compensating for gaps in formal institutional support.

However, recent scholarship increasingly challenges the assumption that such networks function as uniformly supportive communities. Instead, they are better understood as uneven social infrastructures that distribute resources, recognition, and opportunities through relational processes (Dangal and Singh, 2020; Laursen, 2021). Within these infrastructures, access is not equally available but contingent on social evaluation. Under conditions of density, closure, and homophily, visibility is intensified, and information circulates rapidly, amplifying the consequences of inclusion and exclusion (Brouwer et al., 2022; Chen and Beardsley, 2025; Cheng and Liu, 2021; Krajewski et al., 2025; Ma et al., 2021; Nokkala et al., 2022; Wang and Mireles-Rios, 2025).

In such contexts, reputational standing becomes a central organising principle. Rather than relying solely on observable performance, individuals are evaluated through interpretations of relational conduct and propriety, which function as reputational cues guiding inclusion and exclusion (Lamont, 2012; Stock, 2021). Reputation thus operates as a form of social currency that structures access to information, support, and belonging.

Despite this shift, existing research remains limited in explaining how reputational evaluation becomes routinised as a form of informal regulation. This limitation points to the need to conceptualise reputational evaluation not merely as a background condition but as an interactionally produced process through which access to resources is actively regulated. Addressing this gap requires closer attention to the mechanisms through which reputational cues are generated, circulated, and stabilised in everyday interaction.

### 2.2 Relational aggression as a mechanism of informal regulation

Relational aggression provides a critical lens for understanding how reputational processes operate at the level of interaction. It refers to indirect, relationship-based practices such as exclusion, rumour circulation, alliance formation, and manipulation of social ties (Kyranides et al., 2024; Mehari et al., 2018; Owens et al., 2000). While often examined as a form of interpersonal harm, these practices also function as mechanisms of social boundary maintenance.

Rather than isolated behaviours, relational aggression is better understood as a patterned interactional process that reconfigures belonging and access to resources. In this sense, relational aggression does not simply reflect conflict but constitutes a mechanism through which reputational judgements are enacted and stabilised within network relations. Psychological research demonstrates that such processes are associated with peer victimisation and emotional distress (Benenson et al., 2013; García-Fernández et al., 2023; Wei et al., 2026). Importantly, these effects are not merely outcomes but are integral to how informal regulation is internalised and reproduced (Miguel and Santos, 2022; Schacter and Juvonen, 2019).

These dynamics are shaped by broader gendered and cultural norms. Gendered expectations of respectability intensify reputational accountability, rendering women more vulnerable to indirect forms of exclusion (Ajah-Okohu, 2025; Benenson et al., 2013). For Chinese female international students, these processes are further mediated by culturally embedded norms of propriety and face, which heighten sensitivity to evaluation and increase the perceived risks of confrontation (Ma et al., 2021; Zhou and Zhang, 2024; Zhu et al., 2024).

However, existing research has not sufficiently theorised how these interactional practices connect to broader processes of informal regulation. This gap calls for a reconceptualisation of relational aggression not only as interpersonal harm but as a mechanism through which reputational evaluation is operationalised and stabilised across networked interactions.

### 2.3 Institutional recognition and the invisibility of cumulative harm

While peer-based processes shape everyday experiences of inclusion and exclusion, their consequences are mediated by institutional systems that define what counts as recognisable harm. Drawing on institutional theory, formal reporting mechanisms in higher education operate according to logics of incident-based accountability, privileging events that are temporally bounded, individually attributable, and evidentially verifiable (Kennedy et al., 2025; PettyJohn et al., 2023; Sarkar and Shukla, 2023).

However, harms produced through relational aggression are often cumulative, dispersed, and socially distributed. As a result, they do not align with institutional criteria for recognition. Student experiences may therefore be misclassified as interpersonal conflict or dismissed due to lack of evidence (Alowais and Suliman, 2025; Franco-Santos et al., 2017; Jamawadi, 2025; Velthuis et al., 2021). This misalignment reflects a structural legibility gap in which institutional frameworks actively shape the boundaries of recognisable harm (Ajah-Okohu, 2025; Smith and Freyd, 2014).

Relational and reputational processes generate harms that are consequential yet difficult to translate into incident-based categories (Kawabata et al., 2024; Leff et al., 2010; Voulgaridou and Kokkinos, 2023). Consequently, students experience not only peer-directed harm but also institutional non-recognition, which may reinforce withdrawal and constrain help-seeking.

### 2.4 Conceptual framework of moralised reputational governance

**Figure 1:**
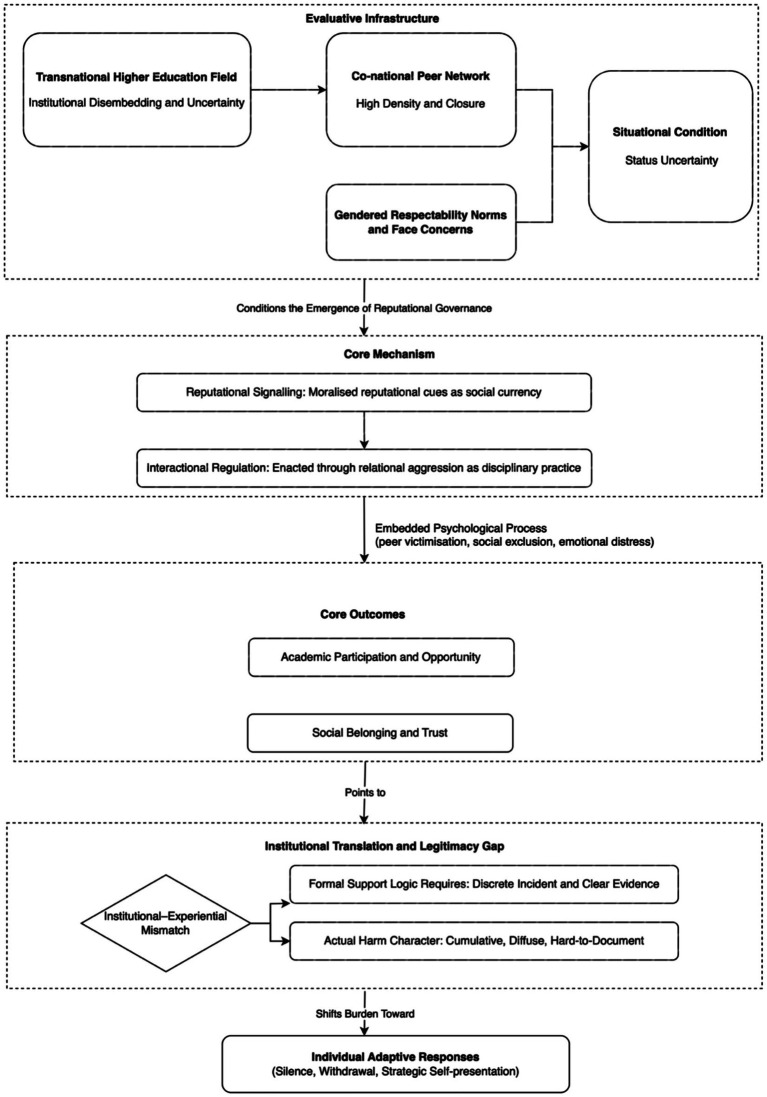
A conceptual framework of moralised reputational governance in co-national peer networks.

Bringing these strands together, this study develops a conceptual framework of moralised reputational governance (see Figure 1) that links interactional mechanisms, network conditions, and institutional logics. Co-national peer networks are conceptualised as evaluative infrastructures in which reputational cues function as organising principles of inclusion and exclusion. Relational aggression operates as the interactional mechanism through which these evaluations are enacted, while institutional systems shape whether and how resulting harms are recognised.

This framework conceptualises peer dynamics as a multi-level process in which evaluation, interaction, and institutional recognition are analytically interconnected rather than sequential. At the interactional level, everyday conduct is moralised and transformed into reputational cues. At the network level, these cues circulate and stabilise through relational ties, organising patterns of inclusion and exclusion. At the institutional level, cumulative harms produced through these processes encounter a legibility gap as they fail to align with incident-based systems of recognition.

By integrating these levels, the framework provides a process-oriented account of how informal governance operates within co-national peer networks. It moves beyond descriptive accounts of ambivalence to specify how evaluation, interaction, and institutional recognition are structurally linked in shaping students’ experiences.

## 3 Methodology

### 3.1 Research design

This study adopts a qualitative interpretive design to examine how Chinese female international students experience and navigate relational aggression within co-national peer networks. It is specifically oriented toward identifying interactional mechanisms rather than measuring prevalence or testing predefined variables. Such an approach is particularly suited to forms of harm that are cumulative, reputational, and embedded in everyday interaction, and that do not readily align with incident-based categories.

The study employs reflexive thematic analysis (Braun and Clarke, 2021a, 2021b), treating participants’ accounts as situated reconstructions of interactional processes rather than objective records of events. This analytic orientation enables attention to how meaning is produced, circulated, and stabilised within relational contexts. The study follows a logic of analytic generalisation rather than statistical inference, aiming to develop theoretically transferable insights into how moralised reputational governance operates across similar conditions.

### 3.2 Participants and sampling

Eight Chinese women with overseas study experience (undergraduate to doctoral levels) were recruited through purposive and snowball sampling via WeChat and related platforms. Participants were eligible if they had experience in co-national peer networks and were able to recall specific interactional episodes. Those without such experience or unable to provide detailed accounts were excluded.

The sample included participants across undergraduate, master’s, and doctoral levels and across multiple host countries, allowing variation in institutional contexts and network conditions. The relatively small sample size is consistent with a mechanism-oriented qualitative design, where the goal is to generate theoretically rich and information-dense accounts rather than achieve statistical representativeness.

Sampling prioritised information-rich cases to support analytic generalisation. Saturation was assessed at the level of interactional mechanisms, with no new patterns of moral coding, reputational cue circulation, or relational sanctioning emerging in later interviews.

### 3.3 Data collection

Semi-structured interviews were conducted in Mandarin via secure video calls lasting 60–90 min. An episodic interviewing approach was adopted to elicit detailed accounts of specific interactional events, including actors, sequences, interpretations, and contextual cues, rather than relying on generalised evaluations or predefined labels such as “bullying.”

Follow-up questions were used to trace how meanings evolved and how participants interpreted others’ actions within specific relational contexts. Particular attention was paid to interactional traces (e.g., group chat dynamics, blocking, selective communication) and the conditions under which participants came to recognise harm.

Interviews were audio-recorded with consent, transcribed verbatim, and anonymised. Transcripts were translated into English with attention to preserving pragmatic meaning and cultural nuance, particularly for context-specific concepts such as *mianzi* (face). Participants were given opportunities to clarify or elaborate on their accounts after the interviews. Table 1 summarises participant characteristics.

**Table 1:** Participant characteristics and composite identifiers.

Respondent	Age at time of incident (years)	Host country	Study level at time of incident	Length of stay in host country at time of incident (years)	Time since incident (years)	Current status
R1–AU–UG	20	Australia	Undergraduate	<1	>1	Undergraduate student
R2–DE–UG	18	Germany	Undergraduate	<1	>1	Undergraduate student
R3–UK–UG	23	UK	Undergraduate	<1	>3	Master’s graduate
R4–AU–MA	25	Australia	Master’s level	2–3	>3	Employed
R5–US–MA	25	United States	Master’s level	>3	>6	Employed
R6–KR–PHD	37	South Korea	Doctoral level	>3	>4	Employed
R7–MY–PHD	36	Malaysia	Doctoral level	<1	>3	Doctoral candidate
R8–MY–PHD	38	Malaysia	Doctoral level	2–3	>2	Doctoral candidate

### 3.4 Data analysis

**Figure 2:**
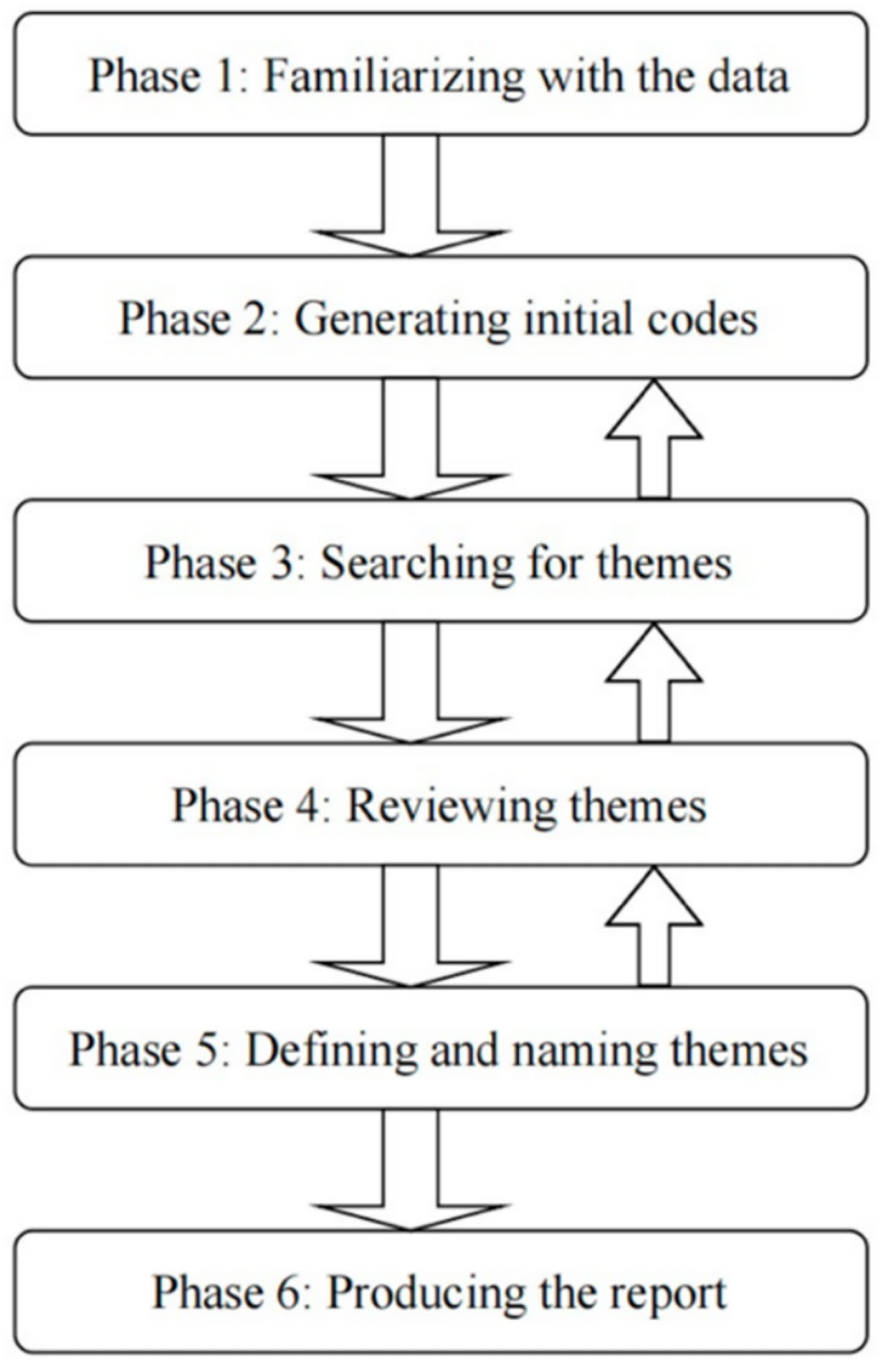
Reflexive thematic analysis: six interactive phases. Adapted from Braun and Clarke (2021a, 2021b).

Data were analysed using reflexive thematic analysis (Braun and Clarke, 2021a, 2021b). As shown in Figure 2, the analysis followed an iterative and recursive process involving familiarisation, initial coding, theme development, refinement, and reporting. These phases were not treated as linear steps but as ongoing movement between data, codes, and emerging interpretations (Byrne, 2022).

Coding proceeded from line-by-line coding to more interpretive coding and identification of patterns of meaning, which were subsequently grouped into higher-order subthemes. Analytic attention was directed toward three interrelated dimensions: (1) interactional practices through which ordinary behaviour was moralised, (2) processes through which reputational cues were circulated and stabilised, and (3) perceived consequences for belonging, participation, and help-seeking.

Rather than aiming for inter-coder reliability, analytic rigour was ensured through reflexive memoing, iterative engagement with the data, and peer debriefing with qualitative researchers familiar with the research context. Analytic memos documented interpretive decisions, alternative readings, and points at which proposed mechanisms were reconsidered or refined. Particular attention was given to divergent accounts, which enabled refinement of the proposed mechanism and identification of its contextual conditions.

The progression from initial codes to higher-order subthemes is illustrated in Table 2, demonstrating how descriptive coding was developed into theoretically informed interpretation.

**Table 2:** Coding trajectory from initial codes to final subthemes (illustrative examples).

Data segment (from participants’ accounts)	Initial line-by-line code	Interpretive code	Final subtheme
“When I started a relationship, a peer labelled me as overly focused on romance and as having ‘bad character,’ which led others to block and avoid me.” (R1–AU–UG)	Being morally judged for ordinary behaviour; labelled as “bad character”	Ordinary frictions moralised as signals of impropriety	Ordinary frictions became sanctionable through moral reframing
“I stayed out of gossip, but people still made claims about me. When I heard accusations I had never been involved in, I was speechless.” (R6–KR–PHD)	Coalition forming; distancing without verification	Coalition alignment and narrative uptake stabilising reputational cues	Coalition alignment, narrative control, and gatekeeping operationalised exclusion
“At first, I thought it was just a misunderstanding. Only later did I realise I was being ignored, but I did not know when it had started.” (R3–UK–UG)	Mistaking exclusion for misunderstanding; unclear onset	Delayed recognition due to dispersed, incremental practices	Harm recognition was delayed because meaning was dispersed and contested
“The more you explain, the darker it gets; explanation is read as ‘creating drama,’ so silence becomes safer.” (R2–DE–UG)	Explanation framed as wrongdoing; deciding to stay silent	Withdrawal as reputational risk management	Silence and withdrawal functioned as reputational risk management
“When I went to the police about the financial issues related to housing, they focused on the contract and unpaid utilities, but the isolation, rumours, and how the story was twisted were basically ignored.” (R4–AU–MA)	Institutional reliance on screenshots/witnesses; denial neutralises reporting	Incident- and evidence-based institutional logic misfitting cumulative harm	Institutional uptake was constrained by incident and evidence logics
“Police procedures could take about 2 years; for doctoral students with research and family duties, it was impossible to pursue.” (R7–MY–PHD)	Excessive procedural timelines; burden shifted to student	Institutional ‘neutrality’ and time delays displacing responsibility	Procedural timelines and neutrality shifted burdens back to participants
“I would cry while eating and even while driving. I was sleeping less than 3 hours a night, and later I was diagnosed with depression and anxiety.” (R5–US–MA)	Severe emotional and embodied distress	Lingering psychological impact and hypervigilance	Psychological distress and vigilance persisted beyond the episode
“I avoided large Chinese circles, lived alone even though it cost more, and kept my distance from others, staying polite but not close.” (R8–MY–PHD)	Avoiding co-national networks; reorganising living/social arrangements	Social contraction as protective reorganisation	Social ties contracted and everyday arrangements were reorganised

### 3.5 Trustworthiness

Trustworthiness was ensured through credibility, dependability, transferability, and confirmability (Enworo, 2023; Rolfe, 2006). Credibility was enhanced through the use of extended participant extracts, episodic detail, and peer debriefing. Dependability was supported by transparent documentation of coding and analytic decisions. Transferability was addressed through detailed contextualisation of participants and settings, allowing readers to assess applicability to other contexts. Confirmability was strengthened through reflexive memoing and explicit engagement with alternative interpretations. Given the interpretive and mechanism-oriented focus of the study, formal member checking was not prioritised; instead, analytic transparency and close alignment between data and interpretation were used to support validity.

This study followed the Consolidated Criteria for Reporting Qualitative Research (COREQ) guidelines (Tong et al., 2007), with a completed checklist provided in Appendix 1.

### 3.6 Reflexivity and ethical considerations

The research team comprised a Chinese female researcher, a Chinese male researcher, and a Pakistani female researcher. This combination of insider and outsider positions enabled both culturally grounded interpretation and critical analytic distance.

The shared linguistic and cultural background of the Chinese researchers facilitated rapport and nuanced interpretation, particularly regarding concepts such as face (*mianzi*) and relational propriety. At the same time, the involvement of a non-Chinese researcher supported critical interrogation of taken-for-granted assumptions and interpretive frameworks.

Reflexivity was treated as an ongoing analytic practice rather than a procedural statement. Reflexive memoing and team discussions were used to examine how positionality shaped data interpretation and to avoid normalising culturally embedded meanings.

Ethical approval was obtained before data collection. Participants provided informed consent, were informed of their right to withdraw or decline questions, and were assured of confidentiality. Data were anonymised and securely stored, with identifying details removed or altered.

## 4 Findings

### 4.1 Relational aggression was enacted through routinised interactional practices in co-national peer networks

#### 4.1.1 Ordinary frictions became sanctionable through moral reframing

Across all eight participants, relational aggression rarely took the form of overt confrontation. Instead, it developed as ordinary interactions were morally reinterpreted. Routine activities such as social invitations (R1–AU–UG; R3–UK–UG), housing arrangements (R4–AU–MA; R6–KR–PHD; R7–MY–PHD; R8–MY–PHD), consumption talk (R2–DE–UG), and academic accountability (R5–US–MA) were later read as signals of flawed character or impropriety.

For R1–AU–UG, beginning a relationship prompted a peer to label her as overly focused on romance and as having “bad character,” leading others to block and avoid her. For R3–UK–UG, inviting one peer to a performance was interpreted as intentional exclusion, triggering coordinated distancing.

Housing interactions were particularly prone to this reframing. R4–AU–MA’s subletting request was reinterpreted as “taking advantage of the apartment.” R8–MY–PHD described minor disputes over utilities accumulating into a sense that “two roommates were aligned against” her. R6–KR–PHD saw routine complaints escalate into attacks on her academic legitimacy, including the claim that she had “paid for a paper rather than wrote it.” R7–MY–PHD experienced repeated mobilisation of small frictions that enabled sequential isolation within a shared lab–housing environment.

Similar reinterpretations occurred in academic and consumption contexts. When R5–US–MA confronted a peer for copying her work, her actions were reframed as “bullying” and “creating conflict.” R2–DE–UG found that casual comments on clothing and jewellery prices were later sexualised into rumours that she was “being financially maintained by a married man.” Across cases, the significance of any single trigger mattered less than its potential to be moralised and circulated as a reputational cue.

#### 4.1.2 Coalition alignment, narrative control, and gatekeeping operationalised exclusion

Once moralised interpretations gained traction, participants described a shared repertoire of practices that enforced exclusion without confrontation. Coalition alignment transformed dyadic tension into collective distancing across cases, raising the cost of dissent and narrowing opportunities for clarification.

R3–UK–UG recalled that peers began “standing on sides,” leaving her uncertain about who had accepted which version of events. R4–AU–MA and R8–MY–PHD similarly described roommates “uniting,” which made exclusion appear as a group consensus rather than a personal dispute. In tightly coupled living-and-working networks, R6–KR–PHD and R7–MY–PHD experienced coalition shifts as particularly destabilising, as relational alignment within housing immediately affected access to academic and social resources.

Narrative control stabilised these alignments by re-authoring targets as morally problematic persons. R1–AU–UG noted that reputational talk generated avoidance even without certainty, explaining that “even if you don’t believe it, you still feel scared talking to that person.” R2–DE–UG similarly observed that people who “didn’t know the situation” nonetheless distanced themselves after hearing rumours. For R6–KR–PHD, reputational claims circulated despite her deliberate refusal to participate in gossip, leaving her “speechless” at encountering accusations she had not engaged with.

Surveillance and gatekeeping further restricted response options. R8–MY–PHD described persistent “prying,” where refusing to share personal information was taken as proof that she was “not sincere,” and fragments were later used to construct rumours. R5–US–MA reported coordinated messages and late-night calls urging her to stop pursuing accountability, framed through collective norms such as “don’t create division.” For R1–AU–UG and R3–UK–UG, exclusion was reinforced by being blocked or refused direct conversation, removing channels through which meaning could be repaired once collective judgment had formed.

### 4.2 Ambiguity and reputational risk shaped interpretation and response

#### 4.2.1 Harm recognition was delayed because meaning was dispersed and contested

Across participants, harm was difficult to recognise as it unfolded because exclusion and reputational damage accumulated through dispersed micro-practices rather than discrete incidents. R3–UK–UG initially interpreted distancing as possible misunderstanding, later realising she was being ignored without being able to identify a clear starting point. R1–AU–UG similarly learned of negative judgements only after access to communication had already narrowed. In housing contexts, R6–KR–PHD and R8–MY–PHD described difficulty distinguishing between “practical conflict” and moral sanction, as everyday disputes gradually took on reputational meanings. For R5–US–MA, the instability of meaning was particularly stark: an attempt to resolve plagiarism shifted abruptly into a situation where s These accounts indicate that recognition was delayed not by lack of awareness, but by the relational production of meaning, which depended on others’ uptake and circulation rather than on observable acts.

#### 4.2.2 Silence and withdrawal functioned as reputational risk management

To limit further exposure, most participants shifted to low-visibility responses once reputational talk began to circulate. When speaking up risked being read as misconduct, they described withdrawal, silence, and selective disengagement as pragmatic tactics.

R1–AU–UG said she stopped explaining after a friend advised her not to “enter the game,” fearing that any clarification could be used against her. R2–DE–UG described responding to sexualised rumours as futile, stating that “the more you explain, the darker it gets.” R3–UK–UG framed disengagement as a moral boundary, refusing to explain herself within an interaction order defined by backstage talk.

Time and life-stage constraints further shaped responses. R4–AU–MA prioritised programme completion, noting that “a master’s is at most two years.” R5–US–MA withdrew from formal escalation as graduation approached. Doctoral participants emphasised trajectory protection: R6–KR–PHD reduced co-national contact to safeguard professional reputation, R7–MY–PHD described disengagement as conserving limited energy due to family responsibilities, and R8–MY–PHD framed retreat as propriety, explaining that “public arguing is not decent,” later choosing to live alone despite higher costs.

### 4.3 Incident- and evidence-oriented institutional processes misrecognised cumulative harms

#### 4.3.1 Institutional uptake was constrained by incident and evidence logics

Participants who sought, considered, or observed institutional responses consistently encountered incident- and evidence-oriented logics that mapped poorly onto cumulative, networked harm. R1–AU–UG approached university support services but struggled to narrate complex emotions and dynamics in English; once the other party denied wrongdoing and there were no screenshots or witnesses that clearly “proved” backstage talk, staff concluded there was “no more evidence,” treating the case as a private dispute. R2–DE–UG, who explored legal routes, was told that formal action required witnesses and documents, even though potential witnesses were “usually unwilling to appear.”

R3–UK–UG experienced programme-level mediation that felt “handled and not handled”: staff acknowledged tension and promoted mutual understanding, yet reputational talk continued unchanged. When R4–AU–MA contacted the police over housing-related financial loss, officers focused on the tenancy contract and liability for unpaid utilities, while coordinated isolation, rumours, and narrative reversal remained largely illegible.

For others, barriers were anticipated rather than directly encountered. R5–US–MA feared that screenshots and website records might be read as evidence of “online bullying” instead of context for collective pressure. R6–KR–PHD and R8–MY–PHD doubted the value of reporting when harms consisted mainly of rumours about academic integrity, subtle behavioural shifts, and diffuse avoidance. R7–MY–PHD cited a peer whose police report produced only a temporary pause in one roommate’s behaviour without addressing the wider pattern of triangulation and distrust. Taken together, these accounts indicate that systems organised around discrete, provable violations struggled to register harms produced through gossip, alignment, and cumulative social withdrawal, even when participants were prepared to come forward.

#### 4.3.2 Procedural timelines and neutrality shifted burdens Back to participants

Time and procedural “neutrality” formed a second set of constraints that made formal routes appear costly or ineffective, shifting responsibility for managing harm back onto students. R5-US-MA’s graduation was less than a month away; she expected hearings and investigations to go beyond her timeline. Pursuing police procedures felt incompatible with completing her degree due to academic pressure and distress. Additionally, R7-MY-PHD recalled a peer being informed that police procedures could take “about two years,” which is much longer than doctoral candidates juggling research and family obligations felt they could handle.

In academic settings, procedural neutrality often worked as quiet deflection. R3–UK–UG recalled staff stressing that “everyone is a student” and urging both sides to “move on,” sidestepping unequal voice and reputational harm. R1–AU–UG and R2–DE–UG were told to focus on their studies, “let it go,” or seek informal reconciliation, which eased institutional pressure but left risk and emotional labour with them.

For others, anticipated burdens were decisive. R4–AU–MA knew she could pursue compensation through landlords or police but concluded this would “waste precious time” and prolong contact with those involved. R6–KR–PHD and R8–MY–PHD weighed lengthy, uncertain procedures against priorities such as graduation, childcare, or mental stability and chose to “pay the money and move out” or reorganise living arrangements at personal cost. Across cases, formal processes protected their timelines by shifting resolution onto students, who responded with withdrawal, private coping, or leaving the space and relationships.

### 4.4 Enduring consequences reshaped wellbeing and social participation

#### 4.4.1 Psychological distress and vigilance persisted beyond the episode

Participants stressed that effects often outlasted the immediate conflict, taking the form of sustained distress, bodily symptoms, and heightened vigilance. R5–US–MA described severe disruption, including crying while eating or driving, sleeping fewer than 3 hours per night, and avoiding campus, and later reported diagnoses of depression and anxiety with suicidal ideation. She said that even at graduation she fixed her gaze on the stage lights to keep from crying. R3–UK–UG similarly linked ostracism to a lingering unease in winter and noted that unexpected reminders could still pull her back to that period.

R1–AU–UG described intense sadness, difficulty studying, and a sense of having no friends, and later sought counselling. She also found it hard to put feelings into English, which added to helplessness. R2–DE–UG reported less severe distress but became more sensitive to status-related remarks and relied on bodily discomfort as an early signal of reputational threat.

Doctoral participants often presented a composed surface alongside deeper tension. R6–KR–PHD initially dismissed the events as almost amusing, but later described people as frightening and kept emotional distance. R7–MY–PHD reported visceral disgust when encountering the person involved and avoided even basic greetings. R8–MY–PHD drew on Confucian distinctions between “gentlemen” and “petty people” to interpret her continued wariness around gossip and dependency in co-national groups. R4–AU–MA, who saw herself as strong, still described financial loss and reputational strain and the need for ongoing self-talk to limit rumination.

Across accounts, relational aggression appeared less as a single episode than as a lingering condition, easily reactivated by reminders and shaping a more guarded way of relating to others.

#### 4.4.2 Social ties contracted and everyday arrangements were reorganised

Over time, participants narrowed their social worlds to limit further exposure. Many reduced involvement in co-national circles, became more selective about trust, and kept interaction low stakes. R3–UK–UG moved from clique-based socialising to a small trusted circle. R1–AU–UG invested more in local friendships and described her approach as not entering the game. R2–DE–UG adopted a polite but not close stance, staying civil while holding back emotional disclosure.

Housing and campus routines were also adjusted for safety. After conflict in shared housing, R4–AU–MA avoided Chinese cliques and stopped what she called ineffective socialising. R6–KR–PHD accepted financial loss to move out for peace of mind. R8–MY–PHD chose to live alone despite the higher rent and treated this as buying psychological space. R5–US–MA avoided places where she might encounter those who labelled her a bully and did not stay connected with her cohort after graduation. R7–MY–PHD kept distance from Chinese peers and described responding politely without pursuing closeness.

Across accounts, this was not social withdrawal in general but a shift toward smaller, more controllable ties. Choices about housing, information sharing, and invitations were shaped by prior reputational harm, reshaping both daily organisation and emotional well-being.

## 5 Discussion

This chapter advances a process-oriented theory of relational aggression as moralised reputational governance within co-national peer networks. It shows that such networks function not only as sources of support but also as informal systems of regulation, in which moralised evaluations and reputational processes shape access to belonging and resources (Ahmad, 2025; Zhong et al., 2020). By integrating interactional practices, network dynamics, and institutional logics, the analysis develops an account of how relational aggression is enacted, interpreted, and unevenly recognised within transnational higher education contexts.

### 5.1 Interpreting the findings

#### 5.1.1 Enactment of relational aggression in co-national peer networks

The findings show that relational aggression is not primarily enacted through overt confrontation but through the moralisation of everyday interactions. Routine situations, including social invitations, housing arrangements, and academic exchanges, are reinterpreted through shared moral vocabularies such as sincerity, propriety, and not causing trouble. In this process, mundane interactions become reputationally consequential signals that circulate within the network. Taken together, these findings indicate that relational aggression operates through the moralisation of ordinary interaction, rather than through discrete interpersonal conflict.

This suggests that the ambivalence of co-national networks (Laursen, 2021; Si et al., 2025) is not an inherent structural property but is produced through interactional processes. Moralisation links situated interpretation to patterned inclusion and exclusion, while reputational cues are actively produced, circulated, and stabilised, extending research on reputational evaluation (Brouwer et al., 2022; Cheng and Liu, 2021). In this sense, the findings extend Lamont (2012) by showing that symbolic boundaries can emerge from situated interactional reinterpretations rather than solely from pre-existing categorical distinctions.

Indirect practices such as coalition formation, narrative control, blocking, and information gatekeeping function as distributed regulatory mechanisms that reshape access to belonging and resources. This reconceptualises relational aggression as a networked and cumulative process of regulation rather than solely an interpersonal form of harm (Owens et al., 2000; Yue and Zhang, 2023). Network density and closure further intensify these dynamics (Krajewski et al., 2025; Wang and Mireles-Rios, 2025), as interpretive ambiguity accelerates reputational consolidation through collective uptake. These dynamics resonate with Foucault’s (1975) account of disciplinary power, in which regulation operates through continuous visibility and evaluative possibility.

#### 5.1.2 Interpretation and response: reputational vulnerability and strategic withdrawal

The analysis indicates that participants’ interpretations are shaped by conditions of reputational vulnerability, in which visibility itself becomes a source of risk. Consistent with research on relational aggression as peer victimisation (Kim et al., 2022; Mehari et al., 2018), harm unfolds gradually through dispersed interactions. However, the findings suggest that this ambiguity is structurally produced through the circulation of reputational cues within dense networks, where meaning depends on collective uptake rather than observable acts.

Withdrawal and silence emerge as strategic responses rather than indicators of cultural passivity. These responses reflect rational adaptations to interactional environments in which attempts at clarification or defence are likely to be reinterpreted as further misconduct. This challenges deficit-oriented interpretations of Chinese international students as conflict-avoidant by situating withdrawal within conditions of reputational precarity.

These dynamics are further shaped by gendered expectations of relational propriety and culturally embedded concerns with face (*mianzi*) (Ma et al., 2021; Zhu et al., 2024). In line with research on gendered peer dynamics (Ajah-Okohu, 2025; Benenson et al., 2013), accountability is unevenly distributed, with those positioned as reputationally vulnerable facing higher interpretive burdens. Importantly, these adaptive responses not only mitigate risk but also contribute to the reproduction of reputational governance by limiting opportunities for contestation.

#### 5.1.3 Institutional recognition and the legibility gap

The findings reveal a structural legibility gap, defined as the misalignment between cumulative, networked forms of harm and institutional systems organised around discrete, evidence-based incidents. While prior research has noted the limits of institutional recognition (Alowais and Suliman, 2025; Velthuis et al., 2021), the present study shows that this misalignment is not merely procedural but epistemic.

Institutional frameworks operate according to logics of incident-based accountability, privileging temporally bounded, individually attributable, and evidentially verifiable events (PettyJohn et al., 2023; Smith and Freyd, 2014). As a result, harms produced through dispersed interactional practices such as gossip, coalition alignment, and reputational circulation remain difficult to register as legitimate forms of injury. Institutions therefore do not simply overlook relational harm but actively shape the conditions under which harm becomes knowable.

This epistemic limitation resonates with the concept of institutional betrayal (PettyJohn et al., 2023; Smith and Freyd, 2014) whereby systems intended to provide protection fail to recognise or respond to harm due to their own structural constraints. It also aligns with broader discussions of epistemic injustice, in which certain experiences are rendered unintelligible within dominant frameworks of recognition. The legibility gap thus reflects a deeper disjunction between relational forms of harm and institutional ways of knowing.

Temporal and procedural constraints further intensify this gap. Lengthy processes, high evidentiary thresholds, and assumptions of neutrality render formal reporting impractical, particularly for students navigating academic pressures and uncertain timelines. Consequently, responsibility for managing harm is displaced onto individuals, who adopt strategies of withdrawal, avoidance, and private coping.

While these dynamics are shaped by culturally specific norms such as face, the underlying logic of dense informal monitoring and reputational regulation may extend to other high-density transnational student networks where institutional safeguards are limited. This suggests that the legibility gap reflects a broader structural feature of transnational higher education contexts rather than a culturally isolated phenomenon.

#### 5.1.4 Consequences for wellbeing and social participation

The findings indicate that relational aggression operates as a temporally extended condition with enduring consequences for well-being and social participation. Consistent with existing research (Kim et al., 2022; Schiff and Lee, 2025), participants reported anxiety, emotional strain, and heightened vigilance. However, these outcomes are embedded within ongoing processes of reputational governance rather than discrete interpersonal episodes.

Participants actively reconfigure their social relationships in response to these dynamics, withdrawing from co-national networks, limiting emotional disclosure, and forming smaller, more controllable ties. These patterns align with research on coping and social adaptation while demonstrating that such responses are shaped by reputational risk and network structure (Ellis et al., 2024; Elyamany and Abdelwahab, 2025; Nazir and Özçiçek, 2023).

The consequences extend beyond psychological well-being into material and spatial domains, including housing arrangements and everyday routines. Taken together, these findings suggest that relational aggression reshapes longer-term patterns of social participation and relational orientation.

### 5.2 Theoretical implications

This study conceptualises relational aggression as moralised reputational governance, operating through three interrelated processes: the moralisation of everyday interaction, the circulation and stabilisation of reputational cues, and the institutional misrecognition of cumulative harm. These processes are mutually reinforcing, linking micro-level interactional practices with meso-level network dynamics and macro-level institutional logics.

This study makes three theoretical contributions. First, it shifts analytical attention from static descriptions of co-national networks to dynamic interactional processes, demonstrating that support and constraint are produced through moralised evaluation rather than inherent structural properties (Laursen, 2021; Si et al., 2025).

Second, it reconceptualises relational aggression as a distributed and cumulative process of regulation. Rather than viewing it solely as indirect interpersonal harm (Owens et al., 2000; Yue and Zhang, 2023), the findings show that relational aggression operates as a networked system through which reputational cues are produced, circulated, and stabilised.

Third, it demonstrates that institutional frameworks do not merely fail to recognise certain harms but actively constitute the boundaries of recognisable experience (Franco-Santos et al., 2017). By privileging discrete and evidence-based incidents (Velthuis et al., 2021), institutions generate epistemic conditions under which relational and cumulative harms remain difficult to articulate.

### 5.3 Limitations and directions for future research

This study adopts a mechanism-oriented and interpretive approach, and several limitations should be acknowledged.

First, the proposed mechanism of moralised reputational governance is most likely to operate in dense, high-closure networks where reputational visibility is amplified. Its dynamics may differ in more diffuse or institutionally mediated contexts, indicating that the findings should be understood as analytically bounded rather than universally generalisable.

Second, self-selection bias may have influenced the sample, as participants with negative experiences may have been more likely to participate. Future research could explore whether similar mechanisms operate in less conflictual contexts.

Third, the reliance on retrospective accounts introduces potential recall bias. Longitudinal or real-time approaches would provide a more fine-grained understanding of how reputational processes unfold over time.

Fourth, the focus on Chinese female international students enables analytical depth but limits transferability. Comparative research across genders, national groups, and institutional contexts would help clarify the conditions under which similar mechanisms emerge.

Fifth, digital environments appear to play a significant role in reputational circulation but were not systematically analysed. Future studies could examine how online platforms shape visibility, amplification, and persistence of reputational cues.

Sixth, cross-national variation in institutional responsiveness was not examined in a systematic way. Comparative institutional analysis could further illuminate how different regulatory regimes shape the emergence and consequences of the legibility gap.

Taken together, these limitations suggest that what is often framed as cultural avoidance among Chinese international students should be understood as a situated response to specific interactional and institutional conditions, rather than as an inherent behavioural trait.

## 6 Conclusion

This study shows that relational aggression in co-national peer networks is best understood as a cumulative process of moralised reputational governance rather than a set of isolated incidents. Ordinary interactions are reinterpreted through shared moral vocabularies, circulated as reputational cues, and enacted through indirect boundary-making practices that redistribute access to information, belonging, and peer-mediated support. These dynamics are shaped by gendered respectability norms and concerns with face, which constrain students’ communicative options and render silence and withdrawal rational responses to reputational risk. At the institutional level, incident-based reporting systems and evidentiary requirements struggle to register harms that were dispersed, ambiguous, and socially stabilised, thereby producing a structural legibility gap between lived experience and institutional recognition. Taken together, the findings position relational aggression not as an interpersonal anomaly but as a form of distributed social regulation embedded within interactional, network, and institutional processes. This reframing highlights how peer relations in transnational higher education function as sites of informal governance, where inclusion, exclusion, and participation are continuously negotiated through moralised and reputationally mediated practices.

## Data Availability

The datasets presented in this article are not readily available because they contain information that could compromise the privacy of research participants. Requests to access the datasets should be directed to the corresponding author.
