# Supplementary material for: Moralised reputational governance in transnational higher education: relational aggression in Chinese female co-national peer networks
Source: Front Psychol. 2026 Jul 1;17:1798940. doi: 10.3389/fpsyg.2026.1798940 (PMC13370576; doi:10.3389/fpsyg.2026.1798940)
Supplement: Supplementary file 1 [file Supplementary_file_1.docx]

**COREQ (Consolidated criteria for Reporting Qualitative Research) Checklist**

| Topic | Item No. | Guide Questions/Description | Author's Responses |
| --- | --- | --- | --- |
| **Domain 1: Research team and reflexivity** | | | |
| **Personal characteristics** | | | |
| Interviewer/facilitator | 1 | Which author/s conducted the interview or focus group? | Yajing Wang and Wenfei Sun |
| Credentials | 2 | What were the researcher’s credentials? e.g. PhD, MD | PhD |
| Occupation | 3 | What was their occupation at the time of the study? | Faculty and PhD Candidate |
| Gender | 4 | Was the researcher male or female? | Mixed: male and female researchers |
| Experience and training | 5 | What experience or training did the researcher have? | Formally trained in qualitative research and reflexive thematic analysis |
| **Relationship with participants** | | | |
| Relationship established | 6 | Was a relationship established prior to study commencement? | Initial rapport established via online communication prior to interviews |
| Participant knowledge of interviewer | 7 | What did participants know about the researcher? | Participants were informed of the research purpose and researcher background |
| Interviewer characteristics | 8 | What characteristics were reported about interviewer? | Both interviewers maintain a reflexive awareness of their shared Chinese cultural background. Given the all-female sample and sensitive topic, potential gender-based dynamics were actively managed by ensuring an empathetic, non-directive interviewing stance during cross-gender interactions. |
| **Domain 2: Study Design** | | | |
| **Theoretical framework** | | | |
| Methodological orientation | 9 | What methodological orientation underpins the study? | Qualitative interpretive design using reflexive thematic analysis |
| **Participant selection** | | | |
| Sampling | 10 | How were participants selected? | Purposive and snowball sampling targeting information-rich cases |
| Method of approach | 11 | How were participants approached? | Recruitment via social media platforms |
| Sample size | 12 | How many participants were in the study? | Eight participants |
| Non-participation | 13 | How many refused or dropped out? | None |
| **Setting** | | | |
| Setting of data collection | 14 | Where was data collected? | Online interviews via secure video calls |
| Presence of non-participants | 15 | Was anyone else present? | No |
| Description of sample | 16 | What are the characteristics of the sample? | Participants were all Chinese female international students. Detailed in Table 1 in 3.3 Data collection |
| **Data collection** | | | |
| Interview guide | 17 | Were questions/prompts provided? | Semi-structured interview guide used |
| Repeat interviews | 18 | Were repeat interviews carried out? | Not conducted |
| Audio/visual recording | 19 | Was recording used? | Audio-recorded with consent |
| Field notes | 20 | Were field notes made? | Analytic memos used; no formal field notes |
| Duration | 21 | What was duration of interviews? | 60–90 minutes per interview |
| Data saturation | 22 | Was data saturation discussed? | Data adequacy was evaluated based on the interpretive depth, ensuring sufficient conceptual richness to address the research questions. |
| Transcripts returned | 23 | Were transcripts returned? | Participants were invited to clarify or elaborate on interview content when needed; transcripts were not formally returned for verification |
| **Domain 3: analysis and findings** | | | |
| **Data analysis** | | | |
| Number of data coders | 24 | How many coders coded the data? | Primary coding was conducted collaboratively by the first two authors, with broader analytical reflection and triangulation involving the third researcher |
| Coding tree | 25 | Was coding tree described? | Provided in Table 2 (coding structure and progression from codes to themes) and Figure 2 (analytical and coding process) |
| Derivation of themes | 26 | Were themes derived or predefined? | Themes developed inductively and iteratively |
| Software | 27 | What software was used? | No qualitative software was used; coding was conducted manually with reflexive memoing and iterative analysis |
| Participant checking | 28 | Did participants check findings? | No participant checking of findings was conducted, as the study prioritised analytical transparency and reflexive interpretation over participant validation |
| **Reporting** | | | |
| Quotations presented | 29 | Were quotations presented? | Extensive participant quotations included in Findings |
| Data consistency | 30 | Was data consistent with findings? | Clear alignment between data excerpts and themes |
| Clarity of major themes | 31 | Were major themes clear? | Major themes clearly presented in Section 4 |
| Clarity of minor themes | 32 | Were minor themes discussed? | Subthemes and variations reported where relevant |
